# Supplementary material for: Phylogenetically informed predictions outperform predictive equations in real and simulated data
Source: Nat Commun. 2025 Jul 3;16:6130. doi: 10.1038/s41467-025-61036-1 (PMC12229690; doi:10.1038/s41467-025-61036-1)
Supplement: Supplementary file 1 — Supplementary Information [file 41467_2025_61036_MOESM1_ESM.pdf]

Supplementary Information for:

Phylogenetically informed predictions outperform predictive equations in real and simulated data

Contents

Supplementary Figures 2
Supplementary Figure 1 2
Supplementary Figure 2 3
Supplementary Figure 3 4
Supplementary Methods 4
Case study 1: Dating the sample of Euarchonta trees from Wisniewski et al 4
Case study 2: Inserting missing taxa into the avian phylogeny 5
Case study 3: Inserting missing taxa into the bush-cricket phylogeny 7
Case study 4: Inserting *Tyrannosaurus rex* into the dinosaur phylogeny 8
Supplementary References 8

Supplementary Figures

Supplementary Figure 1

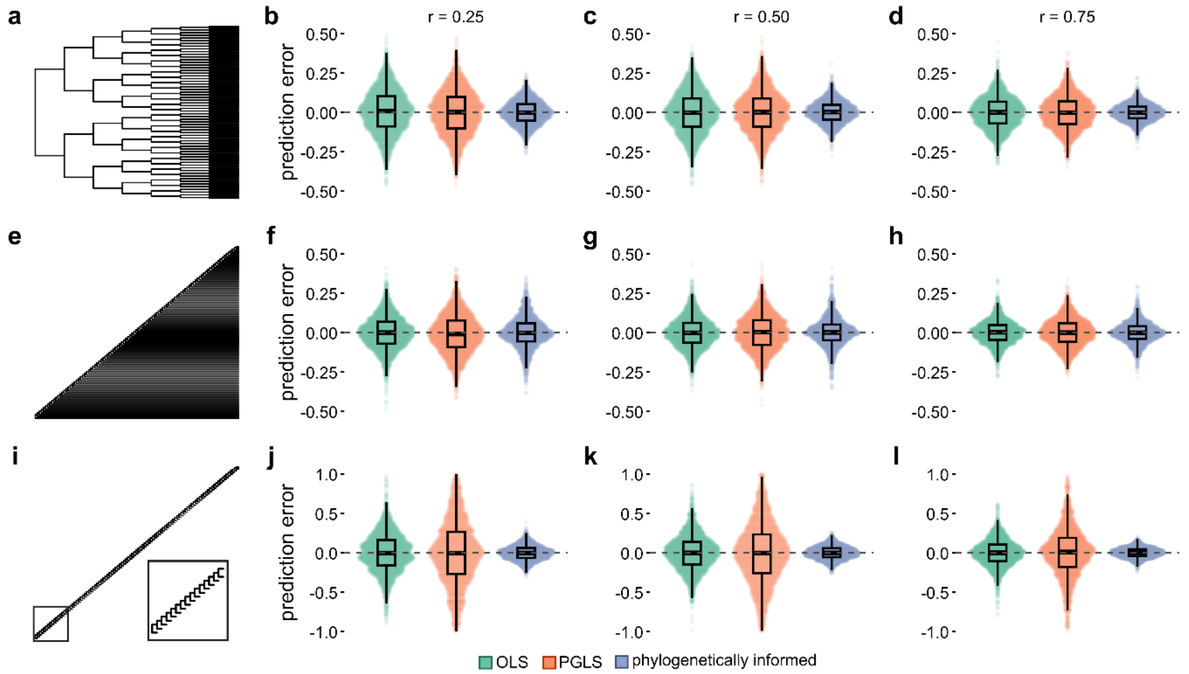

Performance of phylogenetically informed predictions against predictive equations on end-member tree topologies. a-h, Distributions of prediction errors (actual – predicted values) from a balanced ultrametric tree (**a-d**, n = 128 tips) and an imbalanced ultrametric tree (**e-h**, n = 100 tips) under three correlation coefficients (r = 0.25, 0.5, and 0.75). **i-l**, Distributions of prediction errors from a pectinate (ladder-like) tree (n = 100 tips) with inset showing equal branch lengths. Boxplot elements are as follows: centre line, median; box limits, first and third quartiles; whiskers, 1.5 × inter-quartile range; points, outliers. Colours represent the three prediction methods (OLS: ordinary least squares predictive equations, green; PGLS: phylogenetic generalised least squares predictive equations, orange; phylogenetically informed predictions, blue). Tighter distributions near zero indicate more accurate predictions overall than those more skewed away from zero (measured by the variance in prediction errors, σ^2). Phylogenetically informed predictions are more accurate than other methods. PGLS equations are more inaccurate than OLS with pectinate trees. Medians and variances of distributions can be found in Supplementary Data 6.

Supplementary Figure 2

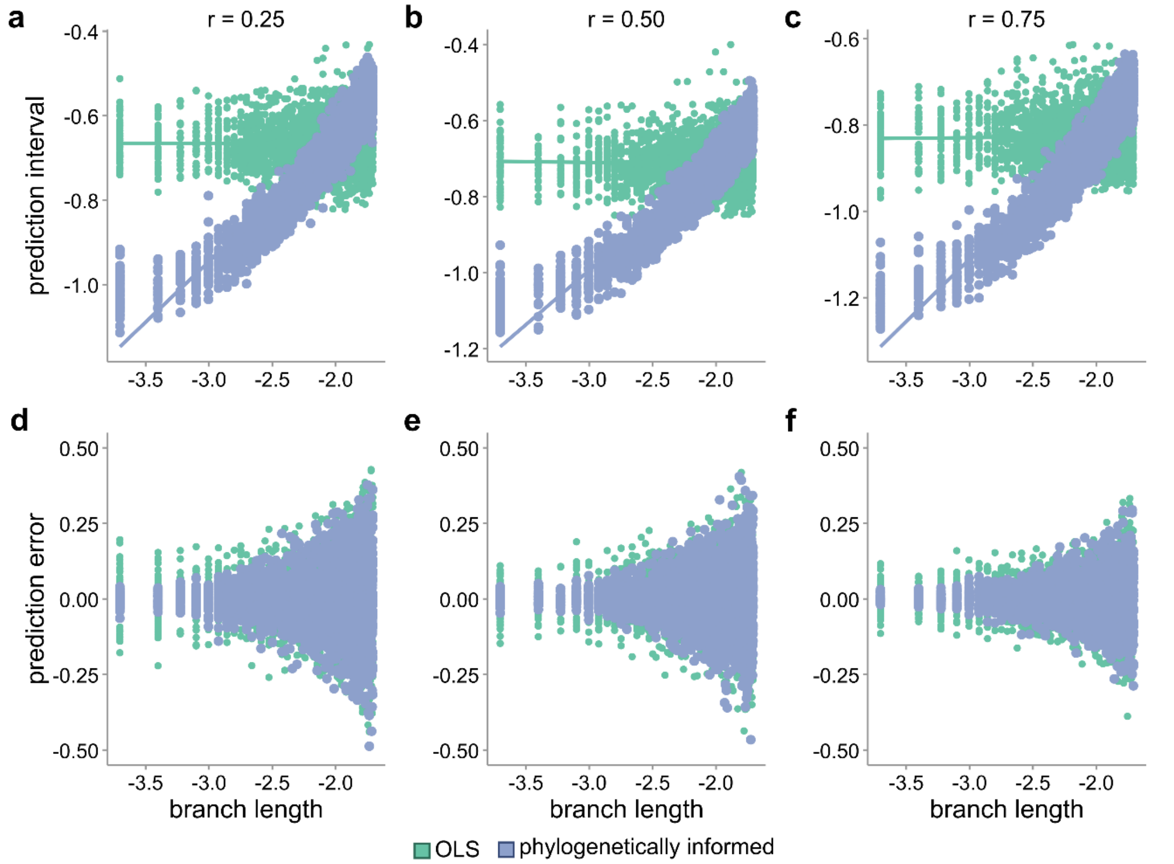

Phylogenetically informed prediction intervals and error increase with terminal branch lengths on an imbalanced ultrametric tree. a-c, Prediction intervals (log_{10}-transformed) of phylogenetically informed predictions (blue points) increase with terminal branch lengths (log_{10}-transformed) of target taxa, whereas ordinary least squares (OLS) prediction intervals do not (green points), on an imbalanced ultrametric tree (see Supplementary Figure 1e) under three correlation coefficients (r = 0.25, 0.50, and 0.75). **d-f**, Range in prediction errors increases with terminal branch lengths (log_{10}-transformed) on an imbalanced tree.

Supplementary Figure 3

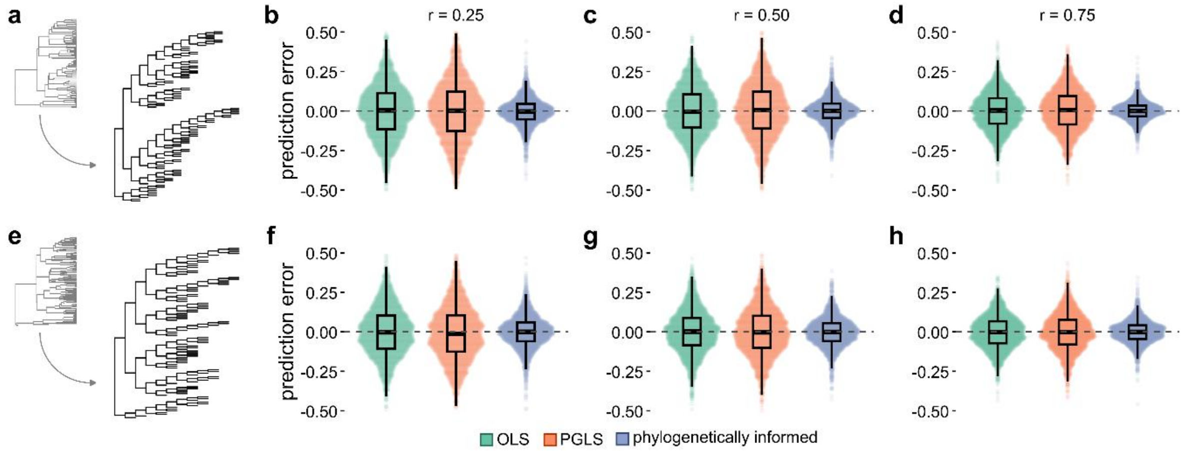

Performance of phylogenetically informed predictions against predictive equations on equal branch-transformed trees. a-d, Distributions of prediction errors (actual – predicted values) from the set of ultrametric trees (see Figure 1a) transformed to equal-length branches under three correlation coefficients (r = 0.25, 0.5, and 0.75). **e-h**, Distributions of prediction errors from the set of non-ultrametric trees (low extinction, Figure 1e) transformed to equal-length branches. Boxplot elements are as follows: centre line, median; box limits, first and third quartiles; whiskers, 1.5 × inter-quartile range; points, outliers. Colours represent the three prediction methods (OLS: ordinary least squares predictive equations, green; PGLS: phylogenetic generalised least squares predictive equations, orange; phylogenetically informed predictions, blue). Tighter distributions near zero indicate more accurate predictions overall than those more skewed away from zero (measured by the variance in prediction errors, σ^2). Phylogenetically informed predictions are more accurate than other methods. Equal branch transformation has little impact on accuracy of prediction methods. Medians and variances of distributions can be found in Supplementary Data 10.

Supplementary Methods

Case study 1: Dating the sample of Euarchonta trees from Wisniewski et al

Here, we use a sample of 100 of the most parsimonious trees from a recently published analysis of Euarchonta[1]. These trees were dated as described in full by Avaria-Llautureo et al. (2024)[2]. However, we provide some detail of how these analyses were conducted here.

The following taxa were excluded owing to broad date ranges and uncertain date placement: Cercopithecini sp. indet. AUH 1321, Colobinae indet. KNM-BN 1251, Colobinae indet. KNM-TH 48368, *Cheracebus purinus*, *Tupaia* sp. UNSM 87244, and Dermoptera indet. Pkg 240 and Pkg 335).

For each of the 100 trees, we fixed the topology and used a fossilised birth-death process[3] implemented in BEAST v2.7[4], conditioning on the root. We used a uniform root calibration prior of between 66 and 130 million years[5] and an optimised relaxed clock. The σ parameter of the log-normal distribution of the clock rates was drawn from an exponential prior with a mean = 1. Transition and transversion rates were drawn from a gamma prior distribution (α = 0.2, β = 0.5) and (α = 0.2, β = 0.25), respectively. A uniform prior distribution ranging between 0 and 1 was

placed on the turnover rate and for the sampling proportion, we used a beta distribution ($\alpha = 5$, $\beta = 90$).

We then calculated a median tree for each of the 100 topologies based on the Kendall-Colijn distance metric[6] using the treespace library[7] in R v.4.0[8].

Case study 2: Inserting missing taxa into the avian phylogeny

In three of the four case studies, it was necessary to insert missing taxa into the phylogenetic tree based on taxonomic affiliation. This can be readily achieved by manually modifying the tree files.

For each of the species in the avian fossil dataset, we identified the closest living relatives in the complete Time Tree of Life[9] based on taxonomic information from the Paleobiology Database (PBDB)[10]. Further details were assessed from published literature where required. We then used the closest existing relative in the tree as an anchor taxon, i.e. a reference point for grafting each taxon. We inserted each taxon into the tree relative to an anchor species or clade at the maximum date of divergence and extended the tip to minimum fossil age measured in millions of years ago (Ma). We inserted species in the tree limited to the species for which we have humerus length data (n = 246) plus a few additional anchor taxa added where necessary (n = 3, see details below). All anchor species were deleted from the tree after placements were completed. Here, we provide the full detail for all taxon placements along with the date ranges for each taxon, consisting of the first and last appearance dates as reported by Crouch et al 2019[11].

The tree including all fossil taxa placed as described below is provided as an additional file (see Supplementary Data 14).

<u>Coraciiformes</u>

There are humerus length measurements for four Eocene Coraciiformes: *Eocoracias brachyptera* (40.4–48.6 Ma), *Primobucco perneri* (40.4–48.6 Ma), *Paracoracias occidental* (45–55 Ma), and *Geranopterus alatus* (33.9–37.2 Ma). These taxa are likely to be basal to or fall within the so-called Coracii – the stem clade including all taxa more closely related to Coracioidea than its nearest outgroup[12,13]. *Coracias* is the only member of Coracioidea included in both our tree and dataset, so this taxon was used to anchor these species to the tree. The branch length for *Coracias* ranges from 0–62 Ma and so all species fall along this branch.

There are two other non-Eocene Coraciiformes with humerus length measurements. The first, *Quasisyndactylus longibrachis,* has recently been highlighted as a fossil needing reassessment, having previously been described as basal to a paraphyletic and now defunct clade – the Alcediniformes[14–16]. However, given modern relationships, and the affiliation of *Quadisyndactylus* to motmots and todies[14], here we treat this species as a Coraciiform. The other, *Brachypteracias langrandi*, is a member of Brachypteraciidae—a family of rollers that is not found in the Time Tree of Life. Both species are therefore placed in a polytomy at the base of the Coraciiform clade.

<u>Zygodactylidae</u>

Eight species with humerus length measurements are identified in the PBDB[10] as belonging to the now extinct family Zygodactylidae, which is considered to be sister to Passeriformes[17,18]:

Primozygodactylus danielsi (48–51 Ma), *P. major* (40.4–48.6 Ma), *P.* ballmanni (48–51 Ma), Pseudastur *macrocephalus* (48–52 Ma), *Eozygodactylus americanus* (50.3–55.8 Ma), *Zygodactylus grandis* (50.3–55.8 Ma), *Z. luberonensis* (28.4–33.9 Ma), and *Serudaptus pohli* (40.4–48.6 Ma). We therefore can place all these species in a polytomy diverging at the maximum observed fossil date for Zygodactylidae (55.8 Ma) along the branch leading to Passeriformes, which ranges between 14 and 66 Ma.

Leptosomiformes

Two species with humerus length measurements are identified in the PBDB as belonging to Leptosomiformes[10] (cuckoo-rollers): *Plesiocathartes kelleri* (51–54.5 Ma) and *Plesiocathartes wyomingensis* (33–45 Ma). We can use the position of this order in the tree to position these taxa as we have no members of this clade included in the extant dataset[19]. To do this, we used the only extant representative of the clade (*Leptosomus discolor)* as an anchor: both fossil species were placed as a sister taxon to the anchor (i.e., within a polytomy), diverging at the maximum age of all members of the clade observed in the tree (55 Ma).

Coliiformes

There are seven extinct Coliiformes (mousebirds) with humerus length measurements: *Masillacolius brevidactylus* (46–52 Ma), *Chascacocolius oscitans* (50–54 Ma), *Eocolius walkeri* (54.4–56.4), *Palaeospiza* bella (33.9–37.2), *Oligocolius psittacocephalon* (24.6–24.8), Coliiformes indet. (45–52 Ma), and *Primocolius sigei* (37.2–40.4 Ma). However, there are no mousebirds in the extant data, so we used a random representative of the clade (*Colius*) to anchor the taxa within this clade. All four species were added as a polytomy falling along the branch leading to *Colius,* which has a branch length ranging between 0–66 Ma.

An additional four species with humerus length measurements are identified in the PBDB[10] as belonging to the extinct family Sandcoleidae: *Eoglaucidium pallus* (48.5–55.8 Ma), *Anneavis anneae* (50.3–55.4 Ma), *Sandcoleus copiosus* (50.3–55.8 Ma), and Sandcoleidae indet. (45–52 Ma). There is reasonably good evidence that this family (sometimes also considered to be a separate extinct order, Sandcoleiformes[10]) falls within or at least sister to extant mousebirds[20–22]. We therefore treated these four species in the same way as mousebirds, inserting them into the polytomy along the branch leading to *Colius*.

Piciformes

Two species with humerus length measurements are identified as Piciformes (woodpeckers and relatives) in the PBDB[10]: *Rupelramphastoides knopfi* (28.4–33.9 Ma) and *Cpitonides protractus* (13.8–16 Ma). There are no members of Piciformes found in the humerus length dataset, and this order is not monophyletic in the Time Tree of Life. We therefore used a random extant representative of the basal-most members of the clade (Coraciimorphae, *Malacoptila fusca)* as an anchor: both fossil species were placed as a sister taxon to the anchor (i.e., within a polytomy), diverging at the maximum age of all members of the clade observed in the tree.

Psittaciformes

Three species with humerus length measurements are identified as Psittaciformes (parrots) in the PBDB[10]: *Cyrilavis olsoni* (48.6–55.8 Ma), *Mopsitta tanta* (48.6–55.8 Ma), and *Messelastur gratulator* (40.4–48.6 Ma). Both *C. olsoni* and *M. tanta* are reportedly basal to the parrot clade[23]. An additional taxon, Pseudasturidae indet. (48.6–55.8 Ma), has also been identified as a stem-group parrot[24]. The root of Psittaciformes in the Time Tree of Life is younger than the observed

fossil ranges and so a polytomy containing all four species was added along the stem branch of this clade.

Upupiformes

Three species with humerus length measurements are identified as falling within the extinct family Messelrrisoridae within Upupiformes (hoopoes and allies) in the PBDB[10]: *Messelrrisor parvus* (40.4–48.6.8 Ma), *M. halcyrostris* (40.4–48.6 Ma), and *M. grandis* (40.4–48.6 Ma). Messelirrisoridae is believed to have been sister to the Upupidae[25].

Another species, *Phirriculus pinicola* (17–22 Ma) is also identified as a member of Upupiformes in the PBDB[10]. This species belongs to the family Phoeniculidae in the PBDB[10,26] which, despite having no representatives in the humerus length dataset, is found in the Time Tree of Life. We therefore used a random representative of this clade, *Phoeniculus purpureus*, as an anchor: *P. pinicola* was placed as a sister taxon at the maximum fossil divergence age.

Todies

Two species have no information about their taxonomic affiliation in the PBDB[10]: *Paleotodus itardiensis* (28.4–33.9 Ma) and *Paleotodus emryi* (33.3–33.9 Ma). However, *Paleotodus* contains the oldest known fossils representing the modern todies (*Todus*)[27]. Here, we use the only *Todus* representative in the dataset, *Todus angustirostris,* as an anchor: both fossil species were placed as a polytomy along the branch leading to this taxon.

Trogoniformes

Primotrogon wintersteini (28.4–33.9 Ma) is assigned to Trogoniformes[10]. We therefore placed this taxon along the branch leading to extant Trogoniformes at the maximum fossil age.

Eufalconimorphae

Psittacopes lepidus belongs to an extinct family of birds (Psittacopedidae), but there is no higher clade information available in the PBDB[10]. Whilst previously considered to be an early parrot, recent phylogenetic analysis has placed this clade as sister to Zygodactylidae (see above) and Passeriformes[28]. To be conservative, we therefore place this taxon in a polytomy at the base of the divergence between parrots (Psittaciformes) and Passeriformes. In the Time Tree of Life, this also happens to include the falcon clade, Falconiformes. The tip was extended to the minimum fossil age (48 Ma).

Case study 3: Inserting missing taxa into the bush-cricket phylogeny

We inserted eighteen taxa into the phylogeny used in our analysis[29] based on the sub-family and tribe-level relationships observed in two more recently published trees of Tettiogoniidae (bush-crickets, Orthoptera: Ensifera)[30], along with the Time Tree of Life[9]. All family and sub-family information was obtained from the NCBI taxonomy browser in combination with the Orthoptera Species File database[31,32]. Below, we describe the taxonomic affiliation for each of the species; in each case they were inserted into a polytomy at the base of the clade representing those groups in the tree we use for analysis.

Several species belong to tribes and families that are individually non-monophyletic but instead fall within a larger clade unifying Mecopodinae, Pseudophyllinae, and Phaneroptidae. This includes the following species: *Mecopoda elongata* (Mecopodini), *Nesonotus vulneratus* and *N.*

caeruloglobus (Cocconotini), *Karukerana aguilari* (Pterophyllini), and *Xerophyllopteryx fumosa*. Additionally, we could find no tribe information for the Phaneropterid species *Philoscirtus viridulus*. All taxa were placed in a basal polytomy at the divergence of the larger clade.

Three species were placed into a polytomy at the base of the monophyletic family group, Conocephalinae, as the tribe they belong to (Copiphorini) was non-monophyletic: *Ruspolia nitidula*, *Acantheremus,* and *Mygalopsis marki*. Additionally, for some tribes within Conocephalinae, only one member was found in the reference trees[31,32]. As most other tribes seem to be non-monophyletic[31,32], these species were also placed within the same polytomy: *Conocephalus nigroopleuron*, *Orchelimum gladiator*, and *Xiphelimum amplipennis* from tribe Conocephalini, and *Afroanthracites* from tribe Agraeciini.

Pseudotettigonia is an extinct genus of insect that was previously assigned to the extant genus *Tettigonia*. We therefore considered members of this genus (*Pseudotettigonia amoena*) to fall within the same tribe as *Tettigonia:* Tettigoniini, again placing within a polytomy.

The species *Aerotegmina taitensis* falls within the sub-family Hexacentrinae, which is sister to the sub-families Meconematinae, Hetrodinae, and Lipotactinae + *Rachidorus* + *Alfredectes* within Tettigoniinae.

We could find no taxonomic information for two tips in the phylogenetic tree: “Amber new gen.” and “*Liliania*”. Both taxa are found within the otherwise monophyletic sub-family Conocephalinae in the tree limited to the species for which we have data. Therefore, these two species were treated as members of this sub-family.

Finally, the fossil species *Archaboilus musicus* does not belong to Tettiogoniidae but instead is a member of an extinct family, Haglidae. We therefore placed this species basal to the entire tree. We chose to not place this species in a polytomy as we have at least some information about the fact that the two groups diverged.

Case study 4: Inserting *Tyrannosaurus rex* into the dinosaur phylogeny

We placed the branch for *Tyrannosaurus rex* along the branch leading to modern birds, diverging at ~216 million years ago, based on the divergence date between *T. rex* and the earliest bird, *Archaeopteryx*, as reported in the maximum clade credibility tree of the recently published dated dinosaur tree by Sakamoto et al[33].

Supplementary References

1. Wisniewski, A. L., Lloyd, G. T. & Slater, G. J. Extant species fail to estimate ancestral geographical ranges at older nodes in primate phylogeny. *Proc. R. Soc. B.* **289**, 20212535 (2022).

2. Avaria-Llautureo, J. *et al.* The radiation and geographic expansion of euprimates through diverse climates. (2024). Preprint available at: doi:https://doi.org/10.32942/X2FS77.

3. Heath, T. A., Huelsenbeck, J. P. & Stadler, T. The fossilized birth–death process for coherent calibration of divergence-time estimates. *Proc Natl Acad Sci U.S.A.* **111**, E2957–E2966 (2014).

4. Bouckaert, R. *et al.* BEAST 2: a software platform for Bayesian evolutionary analysis. *PLoS Comput Biol* **10**, e1003537 (2014).

5. Using Phylogenomic Data to Explore the Effects of Relaxed Clocks and Calibration Strategies on Divergence Time Estimation: Primates as a Test Case. *Syst Biol* **67**(4), 594-615.

6. Kendall, M. & Colijn, C. Mapping Phylogenetic Trees to Reveal Distinct Patterns of Evolution. *Mol BiolE vol* **33**, 2735–2743 (2016).

7. Jombart, T., Kendall, M., Almagro-Garcia, J. & Colijn, C. treespace: Statistical exploration of landscapes of phylogenetic trees. *Mol Ecol Resour* **17**, 1385–1392 (2017).

8. R Core Team. R: A language and environment for statistical computing. R Foundation for Statistical Computing (2023).

9. Hedges, S. B., Marin, J., Suleski, M., Paymer, M. & Kumar, S. Tree of life reveals clock-like speciation and diversification. *Mol Biol Evol* **32**, msv037 (2015) doi:10.1093/molbev/msv037.

10. The Paleobiology Database. https://paleobiodb.org/#/.

11. Crouch, N. M. A. & Mason-Gamer, R. Mass estimation of extinct taxa and phylogenetic hypotheses both influence analyses of character evolution in a large clade of birds (Telluraves). *Proc R Soc Lond B Biol Sci* **286**, 20191745 (2019).

12. Clarke, J. A., Ksepka, D. T., Smith, N. A. & Norell, M. A. Combined phylogenetic analysis of a new North American fossil species confirms widespread Eocene distribution for stem rollers (Aves, Coracii). *Zool J Linn Soc* **157**, 586–611 (2009).

13. Degrange, F. J., Pol, D., Puerta, P. & Wilf, P. Unexpected larger distribution of paleogene stem-rollers (AVES, CORACII): new evidence from the Eocene of Patagonia, Argentina. *Sci Rep* **11**, 1363 (2021).

14. Andersen, M. J., McCullough, J. M., Mauck III, W. M., Smith, B. T. & Moyle, R. G. A phylogeny of kingfishers reveals an Indomalayan origin and elevated rates of diversification on oceanic islands. *J Biogeogr* **45**, 269–281 (2018).

15. Szabo, I. *Kingfisher*. (Reaktion Books, 2019).

16. Mayr, G. The origins of crown group birds: molecules and fossils. *Palaeontology* **57**, 231–242 (2014).

17. Mayr, G. Phylogenetic affinities of the enigmatic avian taxon *Zygodactylus* based on new material from the early Oligocene of France. *J Syst Paleont* **6**, 333–344 (2008).

18. Smith, N. A., DeBee, A. M. & Clarke, J. A. Systematics and phylogeny of the Zygodactylidae (Aves, Neognathae) with description of a new species from the early Eocene of Wyoming, USA. *PeerJ* **6**, e4950 (2018).

19. Field, D. J., Lynner, C., Brown, C. & Darroch, S. A. F. Skeletal correlates for body mass estimation in modern and fossil flying birds. *PLOS ONE* **8**, e82000 (2013).

20. Zelenkov, N. V. & Dyke, G. J. The Fossil Record and Evolution of Mousebirds (aves: Coliiformes). *Palaeontology* **51**, 1403–1418 (2008).

21. Houde, P. & Olson, S. L. A radiation of coly-like birds from the Eocene of North America (AVES: SANDCOLEIFORMES, NEW ORDER). *Natural History Museum of Los Angeles County, Science Series* (1992)

22. Mayr, G. New data on the anatomy and palaeobiology of sandcoleid mousebirds (Aves, Coliiformes) from the early Eocene of Messel. *Palaeobio Palaeoenv* **98**, 639–651 (2018).

23. Ksepka, D. T., Clarke, J. A. & Grande, L. Stem Parrots (Aves, Halcyornithidae) from the Green River Formation and a Combined Phylogeny of Pan-Psittaciformes. *J Paleont* **85**, 835–852 (2011).

24. MAYR, G. On the osteology and phylogenetic affinities of the Pseudasturidae–Lower Eocene stem-group representatives of parrots (Aves, Psittaciformes). *Zool J Linn Soc* **136**, 715–729 (2002).

25. Mayr, G. Tiny Hoopoe-Like Birds From the Middle Eocene of Messel (Germany). *The Auk* **117**, 964–970 (2000).

26. Mayr, G. *et al.* Skeletons from the early Oligocene of Poland fill a significant temporal gap in the fossil record of Upupiform birds (hoopoes and allies). *Historical Biology* **32**, 1163–1175 (2020).

27. Overton, L. C. & Rhoads, D. D. Molecular phylogenetic relationships based on mitochondrial and nuclear gene sequences for the Todies (*Todus*, Todidae) of the Caribbean. *Mol Phyl Evol* **32**, 524–538 (2004).

28. Ksepka, D. T., Grande, L. & Mayr, G. Oldest Finch-Beaked Birds Reveal Parallel Ecological Radiations in the Earliest Evolution of Passerines. *Curr Biol* **29**, 657-663.e1 (2019).

29. Montealegre-Z, F., Ogden, J., Jonsson, T. & Soulsbury, C. D. Morphological determinants of signal carrier frequency in katydids (Orthoptera): a comparative analysis using biophysical evidence of wing vibration. *J Evol Biol* **30**, 2068–2078 (2017).

30. Mugleston, J. D., Naegle, M., Song, H. & Whiting, M. F. A comprehensive phylogeny of Tettigoniidae (Orthoptera: Ensifera) reveals extensive ecomorph convergence and widespread taxonomic incongruence. *Insect Systematics and Diversity* **2**, 5 (2018).

31. Cigliano, M. M., Braun, H., Eades, D. C. & Otte, D. Orthoptera Species File. *Orthoptera Species File* https://orthoptera.speciesfile.org/ (2024).

32. Schoch, C. L. *et al.* NCBI Taxonomy: a comprehensive update on curation, resources and tools. *Database (Oxford)* **2020**, baaa062 (2020).

33. Sakamoto, M., Benton, M. J. & Venditti, C. Strong support for a heterogeneous speciation decline model in Dinosauria: a response to claims made by Bonsor et al. (2020). *Royal Society Open Science* **8**, 202143 (2021).
