# Supplementary material for: Phylogenetically informed predictions outperform predictive equations in real and simulated data
Source: Nat Commun. 2025 Jul 3;16:6130. doi: 10.1038/s41467-025-61036-1 (PMC12229690; doi:10.1038/s41467-025-61036-1)
Supplement: Supplementary file 2 — Description of Additional Supplementary Files [file 41467_2025_61036_MOESM2_ESM.pdf]

# Description of Additional Supplementary Files

**Supplementary Data 1. Fig 1, medians & variances**. Median prediction errors and variances of prediction error distributions for tree shapes in Figure 1 (multiple ultrametric trees, multiple non-ultrametric trees with low extinction, and multiple non-ultrametric trees with high extinction). Performance of predictive equations relative to phylogenetically informed prediction is measured by dividing the variance in errors for predictive equations by the variance in errors for the phylogenetically informed predictions, interpreted as a fold increase in performance.

**Supplementary Data 2. Fig 1, one-sample t-tests**. Intercept-only models on median prediction error differences (absolute PGLS or OLS equation error - absolute phylogenetic prediction error) against a mean of 0 (no difference in error between methods) for tree shapes shown in Figure 1 (multiple ultrametric trees, multiple non-ultrametric trees with low extinction, and multiple non-ultrametric trees with high extinction). A significantly positive estimate for the intercept indicates that phylogenetically informed prediction has lower error (i.e., more accurate) than the predictive equations. These tests compared the phylogenetically informed prediction with PGLS and OLS predictive equations separately.

**Supplementary Data 3. Tree shape tests**. Linear models on log10-scaled absolute prediction errors against tree shape metrics (log10-scaled), including the Colless's and Sackin's balance metrics and stemminess.

**Supplementary Data 4. Tree sizes, medians & variances**. Median prediction errors and variances of prediction error distributions for ultrametric trees and non-ultrametric trees (low extinction) with different tree sizes (n = 50, 250, and 500); results for trees with n = 100 can be found in the sheet "Fig 1, medians & variances".

**Supplementary Data 5. Tree sizes, t-tests**. Intercept-only models on median prediction error differences (absolute PGLS or OLS equation error - absolute phylogenetic prediction error) against a mean of 0 (no difference in error between methods) for ultrametric trees and non-ultrametric trees (low extinction) with different tree sizes (n = 50, 250, and 500). A significantly positive estimate for the intercept indicates that phylogenetically informed prediction has lower error (i.e., more accurate) than the predictive equations. These tests compared the phylogenetically informed prediction with PGLS and OLS predictive equations separately. Results for trees with n = 100 can be found in the sheet "Fig 1, one-sample t-tests".

**Supplementary Data 6. Supp Fig 1, medians & variances**. Median prediction errors and variances of prediction error distributions for tree shapes in Extended Data Figure 1 (balanced ultrametric tree, imbalanced ultrametric tree, and pectinate non-ultrametric tree). Performance of predictive equations relative to phylogenetically informed prediction is measured by dividing the variance in errors for predictive equations by the variance in errors for the phylogenetically informed predictions, interpreted as a fold increase in performance.

**Supplementary Data 7. Supp Fig 1, t-tests**. Intercept-only models on median prediction error differences (absolute PGLS or OLS equation error - absolute phylogenetic prediction error) against a mean of 0 (no difference in error between methods) for tree shapes shown in Extended Data Figure 1 (balanced ultrametric tree, imbalanced ultrametric tree, and pectinate non-ultrametric tree). A significantly positive estimate for the intercept indicates that phylogenetically informed prediction has lower error (i.e., more accurate) than the predictive equations. These tests compared the phylogenetically informed prediction with PGLS and OLS predictive equations separately.

**Supplementary Data 8. High extinct 20%, medians & variances**. Median prediction errors and variances of prediction error distributions for non-ultrametric trees with high extinction and predicting 20% of all taxa.

**Supplementary Data 9. High extinct 20%, t-tests**. Intercept-only models on median prediction error differences (absolute PGLS or OLS equation error - absolute phylogenetic prediction error) against a mean of 0 (no difference in error between methods) for non-ultrametric trees with high extinction and predicting 20% of all taxa. A significantly positive estimate for the intercept indicates that phylogenetically informed prediction has lower error (i.e., more accurate) than the predictive equations. These tests compared the phylogenetically informed prediction with PGLS and OLS predictive equations separately.

**Supplementary Data 10. Supp Fig 3, medians & variances**. Median prediction errors and variances of prediction error distributions for tree shapes in Extended Data Figure 3 (multiple ultrametric trees with equal branch lengths, and multiple non-ultrametric trees with low extinction and equal branch lengths).

**Supplementary Data 11. Supp Fig 3, t-tests**. Intercept-only models on median prediction error differences (absolute PGLS or OLS equation error - absolute phylogenetic prediction error) against a mean of 0 (no difference in error between methods) for tree shapes shown in Extended Data Figure 3 (multiple ultrametric trees with equal branch lengths, and multiple non-ultrametric trees with low extinction and equal branch lengths). A significantly positive estimate for the intercept indicates that phylogenetically informed prediction has lower error (i.e., more accurate) than the predictive equations. These tests compared the phylogenetically informed prediction with PGLS and OLS predictive equations separately.

**Supplementary Data 12. Fig 3, lower quartile medians & variances**. Median prediction errors and variances of prediction error distributions for ultrametric trees and non-ultrametric trees with low extinction, predicting 50% of taxa in the lower quartile of the predicted trait.

**Supplementary Data 13. Fig 3, lower quartile t-tests**. Intercept-only models on median prediction error differences (absolute PGLS or OLS equation error - absolute phylogenetic prediction error) against a mean of 0 (no difference in error between methods) for ultrametric trees and non-ultrametric trees with low extinction, predicting 50% of taxa in the lower quartile of the predicted trait. A significantly positive estimate for the intercept indicates that phylogenetically informed prediction has lower error (i.e., more accurate) than the predictive equations. These tests compared the phylogenetically informed prediction with PGLS and OLS predictive equations separately.

**Supplementary Data 14: This compressed archive file (.zip) contains all tree files and data files for each of the four case studies.** All datasets and trees contain all species, including those with missing data used for inference. There is an index meta-file included in the archive that gives more details.

**Supplementary Code 1: This compressed archive file (.zip) contains all the R code necessary to perform the analysis.** This includes the source code for the phylogenetically-informed prediction method, the code used to generate our simulations at differing correlation coefficients, the code used to summarize the simulation results, the code used to estimate the stemminess metric, and the modified source code used to self-validate the phylogenetically-informed prediction method in Case study 1. There is an index meta-file included in the archive that gives more details.
